# RTS,S Malaria Vaccine and Increased Mortality in Girls

**DOI:** 10.1128/mBio.00514-16

**Published:** 2016-04-26

**Authors:** Sabra L. Klein, Frank Shann, William J. Moss, Christine S. Benn, Peter Aaby

**Affiliations:** a W. Harry Feinstone Department of Molecular Microbiology and Immunology, Johns Hopkins Bloomberg School of Public Health, Baltimore, Maryland, USA; b Department of Paediatrics, University of Melbourne, Melbourne, Victoria, Australia; c Department of Epidemiology, Johns Hopkins Bloomberg School of Public Health, Baltimore, Maryland, USA; d Research Centre for Vitamins and Vaccines, Bandim Health Project, Statens Serum Institute, Copenhagen, Denmark; e Bandim Health Project, Indepth Network, Bissau, Guinea-Bissau

## GUEST EDITORIAL

Malaria was estimated to result in 214 million clinical cases and 438,000 deaths in 2015, primarily in children under 5 years of age. In Africa, malaria causes approximately 10% of all deaths in children under 5 years of age. The RTS,S/AS01 malaria vaccine has been tested in young children in phase III clinical trials and shown to be 18 to 36% efficacious against clinical malaria (1). Although the vaccine may be efficacious against clinical malaria, it does not however reduce overall mortality.

The World Health Organization (WHO) recently published a position paper on malaria vaccines (2), with emphasis on the RTS,S/AS01 vaccine. Although the vaccine has had modest efficacy, the data in Table 1 show that RTS,S was associated with higher all-cause mortality in girls (mortality ratio, 1.91; 95% confidence interval [CI], 1.30 to 2.79; *P* = 0.0006) but not in boys (mortality ratio, 0.84; 95% CI, 0.61 to 1.17; *P* = 0.3343) in both age groups in which the vaccine was tested (i.e., 6 to 12 weeks and 5 to 17 months) (http://www.gsk-clinicalstudyregister.com/files2/9a7b7726-34e2-418d-bea6-c3fb071fd51c). The sex-differential effect is highly significant (*P* = 0.001). There also was a tendency for RTS,S to be associated with a higher risk of fatal malaria in girls (malaria mortality ratio, 1.90 [0.82 to 4.37]) but not in boys (malaria mortality ratio, 1.07 [0.52 to 2.18]). It is counterintuitive that there was no reduction in fatal malaria associated with RTS,S; however, RTS,S was associated with a twofold-higher case fatality ratio in children who got severe malaria (3).

**TABLE 1:** RTS,S malaria vaccine and mortality by sex

Sex and age of group	No. of deaths overall [no. of deaths due to malaria]/no. of persons in group (%)				RTS,S recipient/control risk ratio (95% CI)
	R3R^a^	R3C^b^	R3R and R3C groups combined	C3C^c^	
Males					
5–17 mo	26 [4]/1,509 (1.7)	19 [9]/1,472 (1.3)	45 [13]/2,981 (1.5)	29 [8]/1,471 (2.0)	0.77 (0.48–1.22)
6–12 wk	24 [3]/1,116 (2.2)	26 [8]/1,118 (2.3)	50 [11]/2,234 (2.2)	26 [3]/1,079 (2.4)	0.93 (0.58–1.48)
					
Total			95 [24]/5,215 (1.8)	55 [11]/2,550 (2.2)	0.84 (0.61–1.17)
					
Females					
5–17 mo	35 [9]/1,467 (2.4)	32 [8]/1,500 (2.1)	67 [17]/2,967 (2.3)	17 [4]/1,503 (1.1)	2.00 (1.18–3.39)
6–12 wk	27 [5]/1,064 (2.5)	29 [4]/1,060 (2.7)	56 [9]/2,124 (2.6)	16 [3]/1,100 (1.5)	1.81 (1.04–3.14)
					
Total			123 [26]/5,091 (2.4)	33 [7]/2,603 (1.3)	1.91 (1.30–2.79)

a R3R, 3× RTS,S plus booster RTS,S.

b R3C, 3× RTS,S plus comparator vaccine.

c C3C, controls (comparator vaccines).

**TABLE 2:** Female-male mortality risk ratio in RTS,S malaria vaccine recipients

Age group	Female/male risk ratio (95% CI)		
	R3R^a^	R3C^b^	R3R and R3C combined
5–17 mo	1.38 (0.84–2.29)	1.65 (0.94–2.90)	1.50 (1.03–2.18)
6–12 wk	1.18 (0.69–2.03)	1.18 (0.70–1.98)	1.18 (0.81–1.72)
			
Total			1.33 (1.02–1.74)

a R3R, 3× RTS,S plus booster RTS,S.

b R3C, 3× RTS,S plus comparator vaccine.

The WHO has speculated that the increased mortality in girls was “largely due to the low female mortality in the control arm” and “could be due to chance” (2), despite the *P* value of 0.0006 for girls and a mortality rate after RTS,S of 2.4% in girls compared to 1.8% in boys (risk ratio, 1.33 [1.02 to 1.74]) (Table 2). Although the WHO could be correct in speculating that this finding was due to chance, these numbers suggest a need for caution and additional research. Before RTS,S is introduced into routine vaccination schedules, we should determine whether RTS,S/AS01 increases mortality in girls and investigate possible mechanisms.

There is precedent for the observation that infant girls experience increased mortality following receipt of vaccines. For example, in the 1980s, when the high-titer measles vaccine (HTMV) was introduced to prevent measles in children under 9 months of age, there was a twofold increase in all-cause mortality in girls, but no increase in boys, which led to withdrawal of the vaccine (4). It was subsequently determined that the increased mortality occurred only among girls who received diphtheria-tetanus-pertussis (DTP) vaccine after HTMV and not among girls who received HTMV after their last dose of DTP (5). The interaction between HTMV and DTP may have caused nonspecific negative effects on all-cause mortality in girls but not boys. Evidence from multiple studies of nonlive vaccines, including DTP and the inactivated polio vaccine (IPV), show that these nonlive vaccines have greater detrimental effects for girls than boys (5, 6). Therefore, the increased female mortality after RTS,S/AS01 should not be dismissed as an unexpected finding that occurred by chance. Further clinical studies should explore whether girls need lower doses of the RTS,S/AS01 vaccine or should receive the vaccine with or separately from other vaccines or at different ages than boys.

Preclinical studies in animal models can help provide insights into the biological basis of these observations, but here too, analysis of potential sex effects has been lacking. Published studies of RTS,S or recombinant circumsporizoite protein in mice and nonhuman primates have only reported using adult females or have not reported the sex of the animals (7–9). Generally, in the fields of immunology, vaccinology, and infectious diseases, investigators either do not report the sex of their animals or predominately use female animals (10). This “one size fits all” approach to vaccine research is not working. Preclinical studies should consider how both age and sex affect vaccine responses and outcomes. RTS,S vaccine could also be used to uncover immunological mechanisms for a possible increase in mortality after RTS,S vaccination among girls but not boys.

The RTS,S vaccine is modestly effective at reducing clinical malaria in children, but the sex differences in all-cause mortality should be rigorously studied in both clinical trials and experimental animal models, particularly in light of prior experience with the HTMV. We seek to raise awareness about the need for additional research into how the RTS,S vaccine and, possibly, other vaccines are associated with greater mortality in girls but not boys. This will only be achieved if age and sex are considered in *a priori* hypotheses in vaccine trials to identify and address potential risks early in the vaccine development process.
